# MR Imaging of the Achilles Tendon after Surgical Repair

**DOI:** 10.2174/1874325001711010697

**Published:** 2017-07-31

**Authors:** Kwok Fai Tam, Tun Hing Lui

**Affiliations:** 1 Department of Radiology, North District Hospital, Hong Kong; 2 Department of Orthopaedics and Traumatology, North District Hospital, Hong Kong

**Keywords:** Achilles tendon, Tendon tear, Surgical repair, MRI, Post-operative changes, Scar tissue, Healing

## Abstract

Achilles tendon tear is common and increasingly frequent. Magnetic resonance imaging (MRI) is the modality of choice for radiological evaluation. It is accurate to assess the status and integrity of the tendon with well documented features. In this article, the MR findings of a normal Achilles tendon as well as common diseases like insertional and noninsertional tendinosis, chronic tendinosis with marked lengthening, tendon rupture are illustrated. After a torn Achilles tendon receives surgical repair, it undergoes different stages of healing process including inflammatory, reparative and remodeling phases. Acute scar tissue in the surgical bed may share similar MR features of tendon re-rupture especially in the early healing phase because both are T2W hyperintense. The size of the gap may even appear larger than expected on T2W images possibly due to tendon remodeling. Understanding of the healing process in post-operative period may prevent overestimation of tendon gap and misdiagnosis of re-tear. We describe the MR features of the post-operative changes with serial studies in different months after surgery. The MR findings with the highlights of the expected sequential changes in normal healing process are illustrated in different cases. A case with surgical repair on a partial tear of Achilles tendon is also included.

## INTRODUCTION

Achilles tendon tear is common and increasingly frequent. The best method of treatment is still controversial but surgical treatment tends to decrease tendon re-rupture rate when compared with non-surgical management [1]. The magnetic resonance (MR) features of acute Achilles rupture are well documented [2]. Application of the same MR features in post-operative MR imaging may, however, overestimate the tendon gap and misdiagnose tendon re-rupture. In this article, the MR changes are illustrated in some cases of torn Achilles tendons received surgical treatment.

## DISCUSSION

**Fig.(1A-B):**
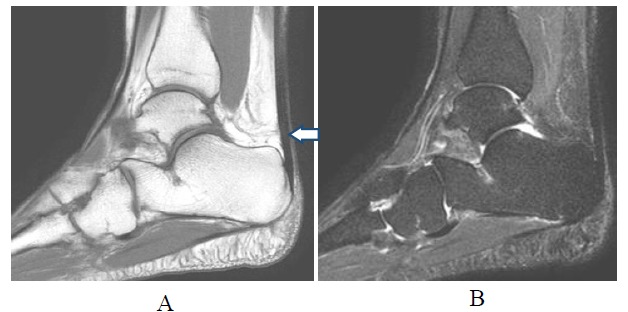
Normal Achilles tendon. T1-weighted and fat suppressed T2-weighted sagittal images show normal hypointense tendon. Small area of internal signal in its distal segment (arrow) may be related to magic angle phenomenon on short TE sequence.

**Fig. (2A-B):**
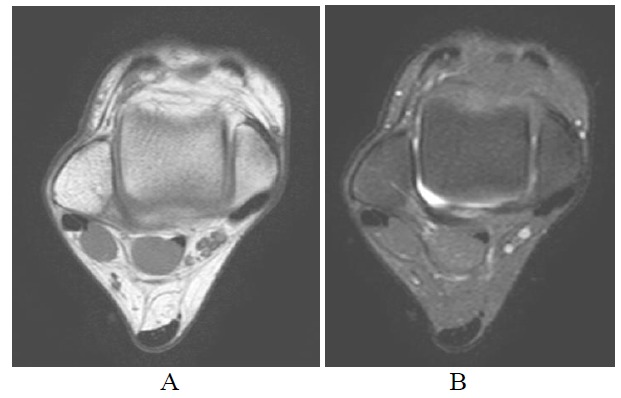
Normal Achilles tendon. T1-weighted and fat suppressed T2-weighted axial images show normal tendon with well defined margin. Its AP dimension should be less than 8mm with concave anterior contour.

MRI has excellent contrast resolution for assessment of the Achilles tendon. Normally, the tendon appears homogeneously hypointense in signal in all imaging sequences. In some asymptomatic cases, small internal signals in its distal segment may be due to artefact related to magic angle phenomenon; not because the tendon itself changes its axis but the internal fibres take spiral twist near the calcaneal insertion (Figs. **1A** and **B**) [2]. The margin of a normal tendon should be smooth and well defined on different orthogonal planes (Figs. **2A** and **B**). It has no tendon sheath but is covered by thin, intermediate signal peritenon. Tendon integrity can easily be assessed on sagittal images, on which the distal end of the calf muscle should be included. Contrast injection usually gives no additional information and plain MR imaging is good enough for assessment. The adjacent structures such as muscle, calcaneus, retrocalcaneal bursa, Kager’s fat pad and subcutaneous fat plane should also be evaluated for associated changes.

**Fig. (3A-B):**
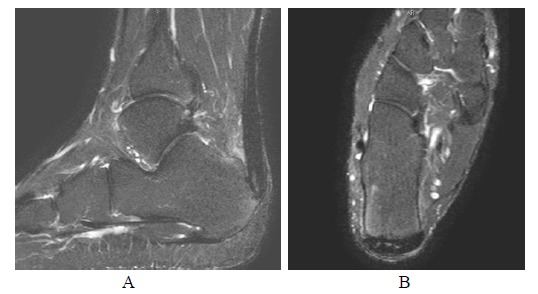
Achilles tendinosis (insertional). Abnormal T2-weighted hyperintense signal is present within the tendon near its insertion.

**Fig. (4):**
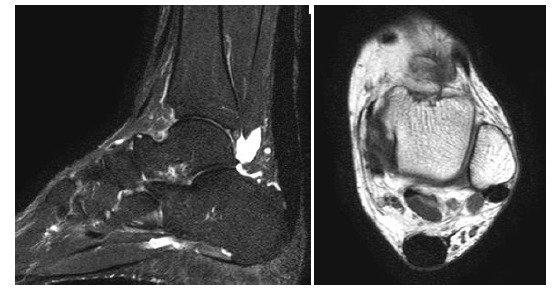
Achilles tendinosis (non-insertional). Achilles tendon is diffusely thickened on sagittal image with minimal internal signal change. Its anterior contour becomes convex on cross section.

**Fig. (5):**
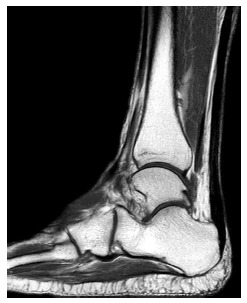
Markedly lengthening of Achilles tendon due to chronic tendinosis.

Similar to other parts of the body, MR diagnosis of a diseased tendon relies on signal intensity and morphologic changes. When a tendon undergoes degeneration, there are usually areas of internal signal changes i.e. T1-weighted intermediate and T2-weighted hyperintense signals within the involved segment. In case of Achilles tendinosis, it shows similar changes with loss of its internal homogeneity and normal hypointense signal, being replaced by T2-weighted hyperintense signal which represents area of mucoid degeneration (Figs. **3A** and **B**). The terms, insertional or non-insertional tendinosis, are used to describe the site of involvement. More commonly, the tendon is thickened with or without internal signal change. It has a bulbous fusiform configuration on sagittal image and its anterior contour becomes convex on cross section (Fig. **4**). Such changes are believed to be the result of hypoxic degenerative tendinosis [3]. In some cases, tendon lengthening with subtle internal signal changes may be rather difficult to be diagnosed by MRI (Fig. **5**).

For partial or complete tear, there will be discontinuity of tendon fibres with a high signal gap, which is filled with blood and edema, on T2-weighted imaging. The proximal tendon end may be retracted in some cases of acute rupture Fig. (**6**). Background signal and morphologic changes of tendinosis are usually present, except those young patients suffering accidental cut injury. Differentiating severe tendinosis from partial tear may sometimes be difficult but those lesions with higher T2-weighted signal intensity are more likely to be tear.

**Fig. (7A-B):**
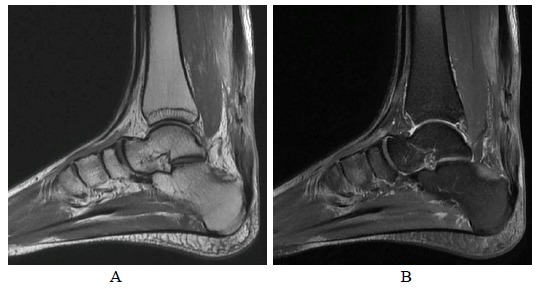
2 months after open surgery for Achilles repair in Patient A. PD-weighted and fat suppressed T2-weighted sagittal images show apparent tendon discontinuity at the operative site. Sutures are present at both ends with no significant separation.

**Fig. (8A-B):**
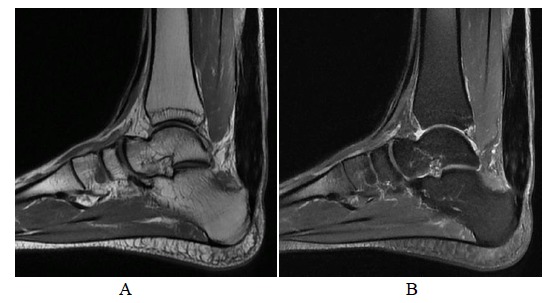
6 months after open surgery for Achilles repair in Patient A. The outline of repaired tendon is now well defined with hypointense scar tissue across the surgical site on PD-weighted and fat suppressed T2-weighted sagittal images.

**Fig. (9A-B):**
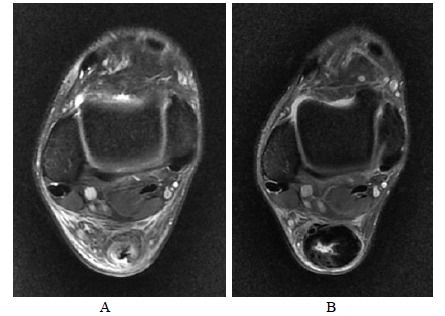
2 months and 6 months after open surgery for Achilles repair in Patient A. Fat suppressed T2-weighted axial images show replacement of internal hypointense scar tissue at the operative site in a centripetal fusion.

The basic principle of surgical treatment for Achilles tendon tear is to bring the torn tendon ends towards each other, inducing the healing process that may take months to complete. If the patient receives surgical repair of the torn Achilles tendon, caution has to be made in the interpretation of early post-operative MR images. The gap and T2-weight hyperintense signal may appear even more extensive when compared with the pre-operative finding. It is well illustrated by Patient A who was suffered from sudden onset of right heel pain during a tennis game. He received an open surgery in a private hospital a day after his injury and was confirmed to be complete Achilles rupture. An early post-operative MRI about two months after his surgery showed ill defined T2 weighted signal with apparent gap across the operative site (Fig. **7**). Such findings are easily misdiagnosed as tendon re-rupture without understanding the sequential changes during the healing process. It was expected that granulation tissue was present across the gap at such early stage. Follow-up imaging six months after the surgery showed disappearance of the gap and replacement of T1- and T2-weighted hypointense fibrous tissue (Fig. **8**). Such changes usually occur in a centripetal fashion, from periphery to centre of the tendon (Fig. **9**).

There are three stages of healing in the injured area of a tendon, including inflammatory stage, reparative stage and finally remodeling stage [4]. The first 24-hour is the inflammatory stage in which white blood cells are accumulated. Angiogenesis and tenocytes proliferation also occur to initiate collagen synthesis that peaks in the next few weeks of reparative stage. The remodeling stage marks the transition of cellular repair tissue to fibrous scar-like tendon tissue. It starts at about 6 weeks and continues even up to a year. Retracted or separated tendon ends do not heal until the torn fibres are brought into close approximation, such that collagenous tissue can proliferate and penetrate the injured area [5].

On MRI, acute scar tissue in the gap appears T2-weighted hyperintense in the first few weeks after surgical repair. The size of the gap usually appears larger than expected during the early post-operative stage. Fujikawa et al. speculated that the tendon gap consists of both the physical gap and remodeling of tendon ends, although they had no histologic proof. Therefore, the size of the gap after surgery may be overestimated on MRI, comparing with the anatomical size of the gap immediately after surgery [6]. The tendon gap is filled with granulation tissue about 7 to 9 weeks after surgery whereas mature fibrous tissue is still minimal. Finally, the gap is expected to disappear approximately 12 weeks after surgery and is eventually replaced by T1- and T2-weighted hypointense fibrotic scar tissue.

**Fig. (10A-B):**
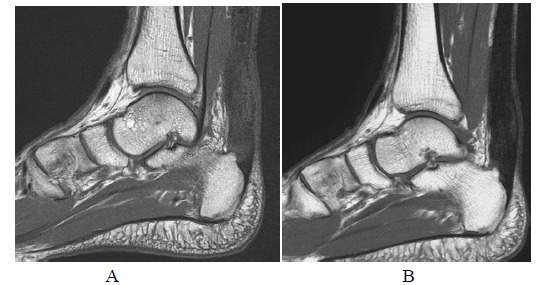
4 months and 12 months after mini-open surgery for Achilles repair in Patient B. The tendon becomes more hypointense and well defined on follow-up scans.

Similar post-operative changes are illustrated in Patient B who received mini-open surgery for Achilles repair. Post-operative MRI (about four months after the surgery) showed better definition of tendon outline with no gapping at the surgical bed. The signal intensity still appeared T1- and T2-weighted intermediate (Fig. **10A**). When the scar matured, the healed tendon became hypointense in all sequences a year after surgery (Fig. **10B**).

Interestingly, the repairing and remodeling process may not be only confined in the physical gap but also happen in the adjacent intact tendon when a tear is incomplete. Patient C was suffered from partial Achilles tear involving the medial half of the tendon which was surgically repaired. Its lateral part of the tendon was confirmed to be intact during the operation. About two months after surgery, T2 weighted signal was present in almost the entire cross section of the tendon, involving both the surgically repaired gap on the medial side and the lateral intact portion of the tendon Fig. (**11A-B**). This can be misinterpreted as complete tear of the Achilles tendon. However, the tendon is intact clinically.

## CONCLUSION

MR evaluation of the Achilles tendon with post-operative changes requires the knowledge of serial changes in different stages of healing response. Caution has to be made to avoid misdiagnosis of tendon re-rupture, especially early after the surgery.

## Figures and Tables

**Fig. (6):**
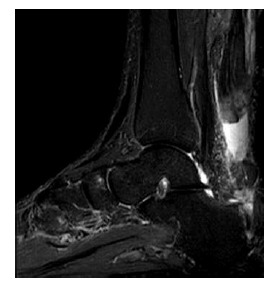
Complete tear of Achilles tendon. The Achilles tendon is completely torn with a gap filled with blood and edema. The proximal end is retracted and thickened.

**Fig. (11A-B):**
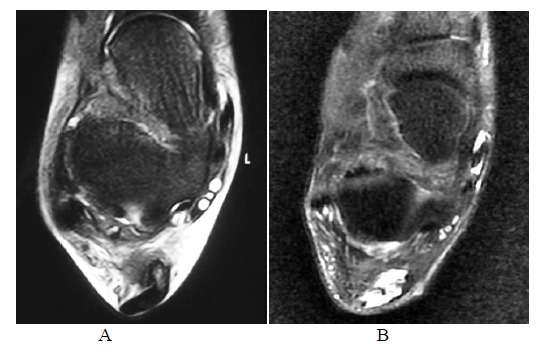
Pre-operative and 2-month post-operative MRI in Patient C with partial Achilles tear. Almost the entire cross sectional area of the Achilles tendon appears T2-weighted hyperintense, even in its previously intact portion.
